# An unusual representation of spider bite with erythema and facial puffiness: a rare clinical image

**DOI:** 10.11604/pamj.2024.47.106.42863

**Published:** 2024-03-05

**Authors:** Yogesh Zamare, Deeplata Mendhe

**Affiliations:** 1 Department of Community Health Nursing, Srimati Radhikabai Meghe Memorial College of Nursing, Datta Meghe Institute of Higher Education and Research, Sawangi (Meghe), Wardha, Maharashtra, India

**Keywords:** Spider bite, unusual representation, envenomation

## Image in medicine

Spider bites are generally considered harmless, with most causing only mild local reactions. In this case, we present a case of a rare and atypical clinical image resulting from a spider bite. During our home visit, we encountered a 68-year-old female in a rural residence with a unique lesion on her face, accompanied by localized pain. She verbalizes to have visited a local clinic 1 week ago for treatment, and she was on medication tablet cetirizine 10mg twice a day, tablet diclofenac 50mg twice a day, and ointment orasore gel 12mg three times a day. Upon examination, multiple areas of redness and facial puffiness, swelling, and erythema are seen that spread on the left side of her face. However, the client reports no improvement in her condition. She was then taken to the hospital for further management and treatment. This case emphasizes the importance of considering uncommon presentations of spider bites in clinical practice. The rarity of such occurrences underscores the need for a comprehensive approach to diagnosis and management. By presenting this rare clinical image, we aim to increase awareness among healthcare professionals, fostering early recognition and appropriate intervention in cases of unusual spider envenomation.

**Figure 1:**
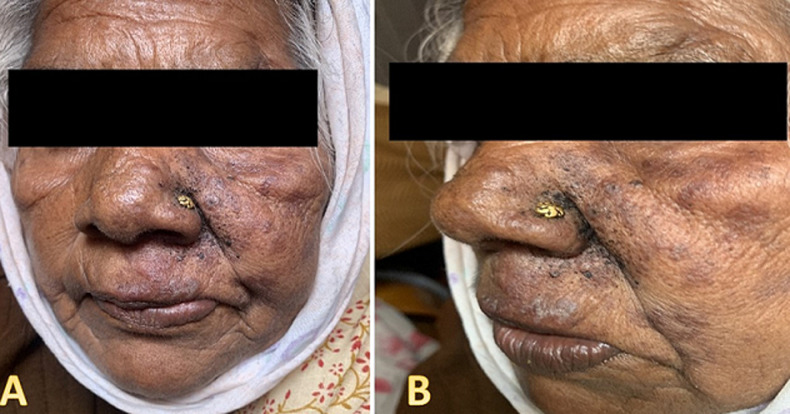
A) frontal view of patients face showing edema, facial puffiness and erythema; B) lateral view of patients face showing black spots and erythema that spreads on the left side of the face

